# Management of primary spontaneous pneumothorax in the pediatric population: a 7-year institutional experience

**DOI:** 10.1590/1806-9282.20260016

**Published:** 2026-08-03

**Authors:** Mehmet Akif Tezcan, Emre Gedik, İbrahim Ethem Özsoy, Oguzhan Turan, Mehmet Akif Ekici, Bayram Metin, Simanur Tezcan

**Affiliations:** 1 University of Health Sciences, Kayseri City Training and Research Hospital, Department of Thoracic Surgery – Kayseri, Türkiye.; 2 Gazi University, Faculty of Medical – Ankara, Türkiye.

**Keywords:** Childhood, Pneumothorax, Bullae, Video-assisted thoracic surgery, BMI

## Abstract

**OBJECTIVE:**

Primary spontaneous pneumothorax is characterized by the accumulation of air within the pleural space in the absence underlying lung diseases. This study aims to evaluate the clinical characteristics, treatment modalities, and recurrence rates of child and adolescent patients diagnosed with primary spontaneous pneumothorax in our clinic.

**METHODS:**

Patients under the age of 18 diagnosed with primary spontaneous pneumothorax via chest X-ray and thoracic computed tomography were included in the study. Patients’ age, gender, presenting complaints, side of pneumothorax, treatment method, complication status, history of recurrence, smoking status, body mass index, and Haller index were recorded. The pneumothorax ratio was calculated radiologically according to the British Thoracic Society guidelines and using the Kircher-Swartzel formula.

**RESULTS:**

Total of 33 patients were included in the study. During follow-up, 15 recurrent episodes occurred, yielding a total of 48 pneumothorax episodes for analysis. No patient experienced more than two total episodes. A statistically significant relationship was identified between the pneumothorax percentage and the development of recurrence. No statistically significant difference was found between body mass/Haller/gender index and the development of recurrence. No significant association was found between the side of pneumothorax (left, right, or bilateral) and the development of either recurrence or complications.

**CONCLUSION:**

Although management strategies similar to adult protocols are applied in childhood pneumothorax, individualized treatment planning based on patient characteristics is of paramount importance. Large-scale studies are required to establish a standardized treatment protocol.

## INTRODUCTION

The term pneumothorax was first defined by Marc Gaspard Itard, while the concept of primary spontaneous pneumothorax (PSP) was introduced to the literature by Kjaergard in 1932^1,2^. PSP is characterized by the accumulation of air within the pleural space in the absence of trauma, invasive procedures, mechanical ventilation, or underlying lung diseases such as pulmonary infection or cystic fibrosis^3,4^.

The incidence of PSP in Taiwan has been reported as 3.4–18 per 100,000 in males and 1.2–6 per 100,000 in females^5^. A large-scale retrospective study conducted in the United States between 1997 and 2006 reported this rate as 2.68–3.41 per 100,000. It is noted that the incidence is higher in the adolescent age group, reaching approximately 10 per 100,000^5,6^. Although the rupture of blebs and bullae is considered the primary mechanism in the development of pneumothorax, the pleural porosity theory is also proposed as an alternative explanation^7^. In children and adolescents, tall stature, low body mass index (BMI), and smoking are among the significant risk factors^8,9^.

Despite increasing interest in pediatric PSP, evidence regarding predictors of recurrence and optimal treatment strategies in children and adolescents remains limited. Most current recommendations are extrapolated from adult populations, and pediatric-specific data are still scarce. In particular, the relationship between pneumothorax size, recurrence risk, and clinical outcomes in pediatric patients has not been sufficiently clarified. Therefore, this study aimed to evaluate clinical characteristics, treatment modalities, recurrence patterns, and potential predictors of recurrence in a pediatric PSP cohort.

## METHODS

This retrospective study was conducted at the Kayseri City Training and Research Hospital, Department of Thoracic Surgery, between January 1, 2018, and January 1, 2025, in accordance with the Declaration of Helsinki. Approval for the study was obtained from the Kayseri Clinical Research Ethics Committee (Date: March 11, 2025, No: 370). Patients under the age of 18 diagnosed with PSP via chest X-ray and thoracic computed tomography (CT) were included in the study. This study included 33 pediatric patients diagnosed with PSP. Among these patients, 15 recurrent episodes developed during follow-up, yielding a total of 48 pneumothorax episodes for analysis. Accordingly, demographic and baseline clinical characteristics were evaluated per patient, while treatment-related variables, episode characteristics, and complications were analyzed per episode. Follow-up duration was calculated from the date of the first pneumothorax episode to the date of the last outpatient visit or last available clinical record. Data were obtained retrospectively through the hospital information system. Patients’ age, gender, presenting complaints, side of pneumothorax, treatment method, complication status, history of recurrence, smoking status, BMI, and Haller index were recorded. The pneumothorax ratio was calculated radiologically according to the British Thoracic Society (BTS) guidelines(A) and using the Kircher-Swartzel formula(B). Treatment decisions and inferential analyses were based on the BTS percentage; Kircher-Swartzel volume was reported descriptively. Oxygen therapy was administered to clinically stable patients (absence of significant dyspnea, tachycardia, hypotension, etc.) with a pneumothorax ratio below 20%, whereas tube thoracostomy was preferred for cases exceeding 20%. Surgical treatment was performed in the presence of an ipsilateral second episode, a first contralateral pneumothorax, a bilateral pneumothorax, or a prolonged air leak. Prolonged air leak was the only clinically relevant complication observed with sufficient frequency to allow analysis; therefore, complication-related comparisons were performed using prolonged air leak as the complication endpoint. All surgical procedures were performed using Video-Assisted Thoracoscopic Surgery (VATS), involving bulla/bleb resection and mechanical abrasion.

Statistical Analysis Statistical Package for the Social Sciences v27.0 (IBM Corp.) software was used for statistical analysis. The distribution of continuous variables was assessed for normality using the Shapiro-Wilk test. Categorical variables were compared using the chi-square test (or Fisher's exact test where appropriate). For comparisons of continuous variables between two groups, the independent samples t-test or Mann-Whitney U test was utilized based on the distribution. A univariable binary logistic regression model was constructed with recurrence as the dependent variable and initial pneumothorax percentage (BTS method) as the predictor. No stepwise selection procedure was used. A p-value of <0.05 was considered statistically significant.

## RESULTS

A total of 33 patients were included in the study. During follow-up, 15 recurrent episodes occurred, yielding a total of 48 pneumothorax episodes for analysis. No patient experienced more than two total episodes. Patient-based demographic and baseline clinical characteristics are presented in Table 1. Among the 33 patients, 81.8% were male, and 18.2% were female, with a median age of 16 years (Q1–Q3: 16–17). The most common presenting complaint was chest pain (78.8%), followed by dyspnea in 21.2% of the patients. The recurrence rate of pneumothorax was found to be 45.5%, with a median interval between two episodes of 4 months (Q1–Q3: 2–12 months). Across the 48 pneumothorax episodes, treatment modalities included tube thoracostomy in 21 episodes (43.8%), VATS bullectomy in 18 episodes (37.5%), and oxygen therapy alone in 9 episodes (18.8%). No conversion to open thoracotomy was required. The median length of hospital stay was 6 days (Q1–Q3: 5–8 days). The median Haller index of the patients was measured as 2.7 (Q1–Q3: 2.46–2.98). The most common complication observed was prolonged air leak (22.9%). No other clinically significant complication was observed at a frequency sufficient for separate analysis. While 48.5% of the patients were smokers, 51.5% were non-smokers. Across the 48 episodes, the median pneumothorax percentage (BTS method) was 38% (Q1–Q3: 21–57), and the median pneumothorax volume (Kircher-Swartzel formula) was 32% (Q1–Q3: 20–53). The median BMI was determined to be 18.7 (Q1–Q3: 17.6–20.8). The median follow-up duration was 41 months (Q1–Q3: 32–54).

**Table 1:** Patient-level and episode-level clinical characteristics of the study cohort.

Variable		n (%)/median (Q1–Q3)
Patient-level characteristics (n=33)		
	Age, years	16 (16–17)
Gender		
	Male	27 (81.8%)
	Female	6 (18.2%)
Presenting complaint		
	Chest pain	26 (78.8%)
	Dyspnea	7 (21.2%)
Recurrence		
	Yes	15 (45.5%)
	No	18 (54.5%)
	Recurrence time, months	4 (2–12)
Smoking status		
	Yes	16 (48.5%)
	No	17 (51.5%)
	Body mass index, kg/m^2^	18.7 (17.6–20.8)
	Haller index	2.7 (2.46–2.98)
	Follow-up duration, months	41 (32–54)
**Episode-level characteristics (n=48)**		
Treatment modality		
	Tube thoracostomy	21 (43.8%)
	VATS bullectomy	18 (37.5%)
	Oxygen therapy	9 (18.8%)
Side of pneumothorax		
	Left	17 (51.5%)
	Right	13 (39.4%)
	Bilateral	3 (9.1%)
	Length of stay, days	6 (5–8)
Complications		
	No prolonged air leak	37 (77.1%)
	Prolonged air leak	11 (22.9%)
	Pneumothorax percentage, % (BTS method)	38 (21–57)
	Pneumothorax volume, % (Kircher-Swartzel formula)	32 (20–53)

VATS: Video-Assisted Thoracoscopic Surgery; BTS: British Thoracic Society.

The clinical and demographic characteristics of the 33 patients (Table 1) and the relationships between these characteristics and the development of recurrence and complications (prolonged air leak) were analyzed (Tables 2 and 3).

**Table 2:** Comparison of patient-level variables according to recurrence status (n=33).

Variable	No recurrence (n=18)	Recurrence (n=15)	p-value
Pneumothorax percentage (BTS method), %	32 (13–38)	55 (23–84)	**0.021**
Body mass index, kg/m^2^	18.05 (17.50–19.60)	19.60 (18.30–22.50)	0.055
Haller index	2.70 (2.45–3.12)	2.80 (2.48–2.98)	0.338
Smoking, yes/no	9/9	7/8	0.849
Gender, male/female	14/4	13/2	0.510
Side, left/right/bilateral	10/8/0	7/5/3	0.137
Follow-up duration, months	38.5 (32–57)	46 (32–54)	0.942

Continuous variables are presented as median (25th–75th percentile), and categorical variables as n (%). Mann-Whitney U test was used for continuous variables. Pearson chi-square was used for categorical variables, as appropriate. A p<0.05 was considered statistically significant. BTS: British Thoracic Society.

**Table 3:** Comparison of episode-level variables according to prolonged air leak status.

Variable		No prolonged air leak	Prolonged air leak	p-value
Pneumothorax percentage*, %		35 (20–47)	55 (30–84)	0.086
Body mass index, kg/m^2^		18.65 (17.60–20.80)	18.70 (16.60–21.20)	0.774
Haller index		2.70 (2.46–2.83)	2.98 (2.46–3.53)	0.118
Gender				
	Male	23	4	0.057
	Female	3	3	
Smoking, yes/no		14/12	2/5	0.470
Side				
	Left	15	2	0.390
	Right	9	4	
	Bilateral	2	1	

* British Thoracic Society guidelines and using continuous variables are presented as median (25th–75th percentile), and categorical variables as counts. Mann-Whitney U test was used for continuous variables. Pearson chi-square was used for categorical variables, as appropriate. Bold values are statistically significant.

A statistically significant relationship was identified between the pneumothorax percentage (BTS method) and the development of recurrence (p=0.021). In binary logistic regression analysis, pneumothorax percentage remained a significant predictor of recurrence (odds ratio=1.037, 95%CI 1.004–1.071, p=0.027; Nagelkerke R^2^=0.228). However, the pneumothorax percentage did not show a significant association with the development of complications (p=0.086) (Tables 2 and 3). No statistically significant correlation was found between the pneumothorax percentage and the length of hospital stay when evaluated using Spearman's rank correlation analysis (r_s_=0.177, p=0.229).

BMI did not reach statistical significance for recurrence (p=0.055). Similarly, no significant difference was observed regarding the development of complications (p=0.774). In both cases, BMI was not considered an independent predictor (Tables 2 and 3).

There was no significant relationship between the Haller index and the development of recurrence (p=0.338). No statistically significant association was found between Haller index and recurrence or complication development (Tables 2 and 3).

No statistically significant difference was found between smoking status (during the episode and post-episode) and the development of either recurrence or complications (p>0.05). Smoking was not considered to have a significant effect on disease recurrence or the development of complications (Tables 2 and 3).

No significant relationship was found between gender and the development of recurrence (p>0.05). Likewise, no difference was observed between male and female patients regarding the development of complications (Tables 2 and 3).

No significant association was found between the side of pneumothorax (left, right, or bilateral) and the development of either recurrence or complications (p>0.05). However, higher complication rates were observed in a small number of bilateral cases (Tables 2 and 3). The relationship between the side of the pneumothorax and the treatment modality applied was also evaluated. Due to the fact that expected values in a large portion of the cells were below 5, the Fisher–Freeman–Halton exact test was used, and no statistically significant relationship was found between the side and the type of treatment (p=0.127).

**Figure 1:**
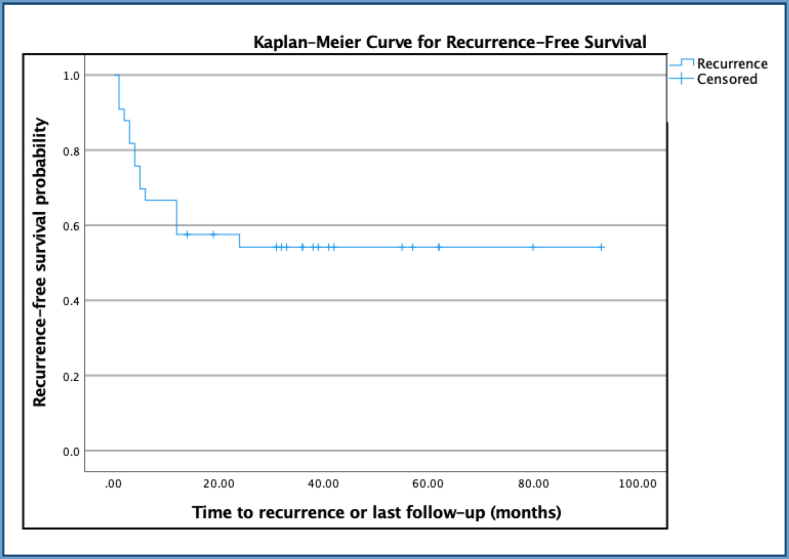
Kaplan-Meier curve for recurrence-free survival in pediatric patients with primary spontaneous pneumothorax. Censored observations are indicated by tick marks.

Kaplan-Meier analysis was performed to evaluate recurrence-free survival. During follow-up, 15 of 33 patients developed recurrence, while 18 patients were censored. The recurrence-free survival probability was 66.7% at 6 months, 57.6% at 12 months, and 54.2% at 24 months. Median recurrence-free survival was not reached during the observation period (Figure 1).

## DISCUSSION

Diagnosis and treatment approaches for PSP in childhood differ from those in adult populations, and a standardized consensus has yet to be established. Current guidelines for pediatric PSP are largely derived from adult PSP protocols, which creates challenges in clinical practice^10^. Consequently, the management of PSP in children varies significantly, and treatment decisions are often based on clinical experience rather than established pediatric-specific guidelines.

Risk factors for the development of PSP include age, gender, body habitus, tall stature, and conditions such as Marfan syndrome^10^. In the literature, pectus excavatum has been identified as a potential risk factor for PSP^11^. Furthermore, female gender and low BMI have been transiently associated with PSP recurrence^12^. One study identified female gender, operated pectus excavatum, age under 12, and a history of contralateral pneumothorax as risk factors for PSP^13^.

In the literature, cases of PSP are often seen in tall, thin, and young males^14^. In our study, no statistically significant relationship was found between gender and recurrence or complications (p=0.51, p=0.057).

Many previous pediatric studies have reported inconsistent relationships between pneumothorax size and the risk of recurrence^15,16^. In our study, a statistically significant relationship was identified between the pneumothorax percentage (BTS method) and the development of recurrence (p=0.021). The role of trans-thoracic drainage in PSP is to remove excess intrapleural air and promote apposition of the visceral and parietal pleura, initiating the process of healing of the lung defect^17^. This finding may be explained by incomplete healing. Therefore, this may support the idea that surgical intervention should be considered earlier in patients with a high percentage of pneumothorax.

The literature has shown that PSP cases are mostly found in tall adolescent boys^14^. Several investigations have suggested that lower BMI and delayed puberty could be risk factors for recurrence, and these findings are consistent with the results of this study^18^. In our study, PSP was more prevalent among male patients. No statistically significant difference was found between BMI and the development of recurrence or complications (p=0.055, p=0.072). In both scenarios, BMI was not considered an independent predictor. Considering the small sample size, this finding may suggest a trend rather than a definitively absent association.

The Haller index demonstrates strong predictive value for recurrent pneumothorax in patients diagnosed with PSP. This finding suggests that the Haller index could be a useful tool for predicting the risk of pneumothorax recurrence in PSP^19^. Although recent studies suggested a predictive role for Haller index in PSP recurrence or complication, no statistically significant association was demonstrated in our pediatric cohort (p=0.338, p=0.118). Higher Haller index values appeared to be associated with an increased rate of complications, although this trend did not reach statistical significance. Therefore, this finding should be interpreted cautiously, particularly given the small sample size and age-related thoracic anatomical differences.

The risk of PSP recurrence increases due to pathophysiological changes in the respiratory system resulting from smoking^20^. In our study, however, no statistically significant difference was found between smoking status (n=16, 48.5%) and the risk of recurrence and complication. Smoking reached its peak in the 16-year-old age group in our cohort; however, we believe the lack of statistical significance may be due to the relatively short duration of smoking exposure in this age group.

In one study in the literature, no statistically significant difference was identified regarding the pediatric PSP aspect^14^. In our study, no significant association was found between the side of pneumothorax (left, right, or bilateral) and the development of either recurrence or complications (p>0.05).

The treatment of the first episode in pediatric PSP remains controversial. The BTS guidelines recommend needle aspiration as the primary treatment for symptomatic PSP. However, one study noted that needle aspiration is ineffective in childhood PSP^21^. The American College of Chest Physicians guidelines recommend a more aggressive approach (tube thoracostomy) for all pneumothoraces exceeding 20%. Another study suggests oxygen therapy for small asymptomatic pneumothoraces and tube thoracostomy for symptomatic patients^22^. Conversely, some studies have reported successful outcomes with primary surgical treatment^23^. In our study, while oxygen therapy was preferred for asymptomatic cases with small pneumothorax volumes, tube thoracostomy was the initial preference for the majority of cases (21 episodes).

A prolonged air leak remains one of the most important indications for surgical intervention in pediatric PSP. While surgery was performed for patients with recurrent or contralateral pneumothorax and prolonged air leak, no patient in our series underwent direct (primary) surgery. Prolonged air leak is defined in various studies as lasting between 3 and 10 days^24^. The ACCP considers air leaks lasting more than 4 days in adults and more than 3 days in children as "prolonged"^24,25^. In our study, we defined air leaks exceeding 5–7 days as prolonged. Eleven patients presenting with prolonged air leak underwent VATS. No major postoperative complications or mortality was observed following surgical intervention in the patient cohort.

The recurrence rate after the first episode in childhood (30–60%) is higher than in adult PSP, independent of the initial pneumothorax size^26^. In our study, this rate was 45.5% (n=15).

Some studies report that childhood itself is a risk factor for PSP recurrence after VATS^27,28^. This is attributed to the fact that the rib cage develops rapidly in children and adolescents, while the lungs develop more slowly^28^. Therefore, pleurodesis may be utilized to prevent recurrence in patients undergoing VATS. Pleurodesis can be performed mechanically (pleurectomy), chemically [talc, OK-432 (Picibanil), or minocycline], or as an autoimmune procedure (autologous blood patch). Chemical pleurodesis induces a local and generalized inflammatory reaction, often accompanied by fever and chest pain; notably, fever may facilitate the pleurodesis process^29^. One study reported a 5% recurrence rate with intraoperative OK-432 pleurodesis^30^, while another study using OK-432 and minocycline reported a recurrence rate of 3.4%^31^. Mechanical pleurodesis typically involves perioperative apical pleurectomy, while the blood patch technique utilizes the patient's own blood.

Chemical pleurodesis, especially the talc group, also has the issue of possible oncogenic risk and difficulty in performing future thoracoscopy or thoracotomy caused by the creation of tight adhesions^32-34^. Clinically, we generally avoid chemical pleurodesis due to concerns regarding potential future thoracic surgeries; however, we prefer pleurodesis via blood patch in cases where a postoperative air leak is excessive.

In one study in the literature, no statistically significant association was found between chest tube duration and recurrence^14^. The median length of hospital stay in our study was 6 days (Q1–Q3: 5–8 days). No statistically significant relationship was found between the duration of hospitalization and the pneumothorax percentage or side.

## CONCLUSION

Although management strategies similar to adult protocols are applied in childhood pneumothorax, individualized treatment planning based on patient characteristics is of paramount importance. Among the methods used in treatment, we consider chemical pleurodesis unsuitable due to its potential impact on future thoracic interventions. Prospective multicenter pediatric studies are needed to establish evidence-based and standardized management algorithms for pediatric PSP.

### Limitations of the study

This study has several limitations. First, its retrospective single-center design and relatively small sample size may limit the generalizability of the findings. Second, treatment decisions were based on institutional clinical practice rather than standardized pediatric-specific protocols. Third, due to the limited number of recurrence and complication events, extensive multivariable analyses could not be performed. Nevertheless, the study provides additional pediatric-specific data regarding recurrence patterns, treatment strategies, and outcomes in pediatric PSP patients.

## Data Availability

The datasets generated and/or analyzed during the current study are available from the corresponding author upon reasonable request.

## References

[1] Henry M, Arnold T, Harvey J (2003). Pleural Diseases Group, Standards of Care Committee, British Thoracic Society. BTS guidelines for the management of spontaneous pneumothorax. Thorax.

[2] Isitmangil T, Balkanli K, Yüksel M, Kalayci G (2001). Gögüs cerrahisi.

[3] Fleisher GR, Ludwig S (2010). Textbook of pediatric emergency medicine.

[4] Frechette E, Guidolin K, Seyam A, Choi YH, Jones S, McClure JA (2016;). Identifying primary spontaneous pneumothorax from administrative databases: a validation study. Can Respir J.

[5] Huang YH, Chang PY, Wong KS, Chang CJ, Lai JY, Chen JC (2017). An age-stratified longitudinal study of primary spontaneous pneumothorax. J Adolesc Health.

[6] Dotson K, Timm N, Gittelman M (2012). Is spontaneous pneumothorax really a pediatric problem? A national perspective. Pediatr Emerg Care.

[7] Radomsky J, Becker HP, Hartel W (1989). Pleural porosity in idiopathic spontaneous pneumothorax. Pneumologie.

[8] Ghisalberti M, Guerrera F, Vico A, Bertolaccini L, Palma A, Fiorelli A (2020). Age and clinical presentation for primary spontaneous pneumothorax. Heart Lung Circ.

[9] Shih CH, Yu HW, Tseng YC, Chang YT, Liu CM, Hsu JW (2011). Clinical manifestations of primary spontaneous pneumothorax in pediatric patients: an analysis of 78 patients. Pediatr Neonatol.

[10] Sudduth CL, Shinnick JK, Geng Z, McCracken CE, Clifton MS, Raval MV (2017). Optimal surgical technique in spontaneous pneumothorax: a systematic review and meta-analysis. J Surg Res.

[11] Kılıçgün A, Yakşi O, Ünal M (2019;). Is pectus excavatum a risk factor for spontaneous pneumothorax? "Haller ındex measurements in patients with primary spontaneous pneumothorax. Can Respir J.

[12] Tan J, Yang Y, Zhong J, Zuo C, Tang H, Zhao H (2017). Association between BMI and recurrence of primary spontaneous pneumothorax. World J Surg.

[13] Wu Y, Yu J, Peng Y, Chen C, Zhang N, Zeng Q (2025). Survival analysis of primary spontaneous pneumothorax in children treated with thoracoscopy: a single-center experience. Pediatr Surg Int.

[14] Sarıkaş NG, Sogut SE, Varlıklı O (2024). Outcomes of primary spontaneous pneumothorax in adolescents. Kocaeli Med J.

[15] Engwall-Gill AJ, Morgan K, Weller JH, Gomez-Quevedo O, Bonitto S, DeRosa J (2026). Risks for recurrence after pediatric primary spontaneous pneumothorax: results from a multicenter study. J Surg Res.

[16] Lewit RA, Tutor A, Albrecht A, Weatherall YZ, Williams RF (2021). Pediatric spontaneous pneumothorax: does ınitial treatment affect outcomes?. J Surg Res.

[17] Tsai TM, Lin MW, Li YJ, Chang CH, Liao HC, Liu CY (2017). The size of spontaneous pneumothorax is a predictor of unsuccessful catheter drainage. Sci Rep.

[18] Wilson PM, Rymeski B, Xu X, Hardie W (2021). An evidence-based review of primary spontaneous pneumothorax in the adolescent population. J Am Coll Emerg Physicians Open.

[19] Aydın ÖF, Tatlıparmak AC (2025). Predicting recurrence in primary spontaneous pneumothorax: the role of the Haller index in emergency department patients. Ulus Travma Acil Cerrahi Derg.

[20] Cheng YL, Huang TW, Lin CK, Lee SC, Tzao C, Chen JC (2009). The impact of smoking in primary spontaneous pneumothorax. J Thorac Cardiovasc Surg.

[21] Soccorso G, Anbarasan R, Singh M, Lindley RM, Marven SS, Parikh DH (2015). Management of large primary spontaneous pneumothorax in children: radiological guidance, surgical intervention and proposed guideline. Pediatr Surg Int.

[22] Young Choi S, Beom Park C, Wha Song S, Hwan Kim Y, Cheol Jeong S, Soo Kim K (2014). What factors predict recurrence after an initial episode of primary spontaneous pneumothorax in children?. Ann Thorac Cardiovasc Surg.

[23] Miscia ME, Lauriti G, Lisi G, Riccio A, Lelli Chiesa P (2020). Management of spontaneous pneumothorax in children: a systematic review and meta-analysis. Eur J Pediatr Surg.

[24] Williams K, Baumann L, Grabowski J, Lautz TB (2019). Current practice in the management of spontaneous pneumothorax in children. J Laparoendosc Adv Surg Tech A.

[25] Robinson PD, Cooper P, Ranganathan SC (2009). Evidence-based management of paediatric primary spontaneous pneumothorax. Paediatr Respir Rev.

[26] Butterworth SA, Blair GK, LeBlanc JG, Skarsgard ED (2007). An open and shut case for early VATS treatment of primary spontaneous pneumothorax in children. Can J Surg.

[27] Choi SY, Kim YH, Jo KH, Kim CK, Park JK, Cho DG (2013). Video-assisted thoracoscopic surgery for primary spontaneous pneumothorax in children. Pediatr Surg Int.

[28] Wilson PM, Rymeski B, Xu X, Hardie W (2021). An evidence-based review of primary spontaneous pneumothorax in the adolescent population. J Am Coll Emerg Physicians Open.

[29] Ukale V, Agrenius V, Widström O, Hassan A, Hillerdal G (2004). Inflammatory parameters after pleurodesis in recurrent malignant pleural effusions and their predictive value. Respir Med.

[30] Huang H, Lin YH, Chang PC, Wang NL, Sheu JC (2022). Intraoperative OK-432 pleurodesis for preventing recurrence of primary spontaneous pneumothorax in children and adolescents: a single-center experience. Pediatr Surg Int.

[31] How CH, Tsai TM, Kuo SW, Huang PM, Hsu HH, Lee JM (2014). Chemical pleurodesis for prolonged postoperative air leak in primary spontaneous pneumothorax. J Formos Med Assoc.

[32] Gossot D, Galetta D, Stern JB, Debrosse D, Caliandro R, Girard P (2004). Results of thoracoscopic pleural abrasion for primary spontaneous pneumothorax. Surg Endosc.

[33] Stewart S, Fraser JA, Rentea RM, Aguayo P, Juang D, Fraser JD (2023). Management of primary spontaneous pneumothorax in children: a single ınstitution protocol analysis. J Pediatr Surg.

[34] Chan IC, Lee YS, Chuang CM, Soong WJ (2019). The influence of pleurodesis on the outcome of primary spontaneous pneumothorax in children. J Chin Med Assoc.

